# Differential diagnosis of pathological intracranial calcifications in patients with microcephaly related to congenital Zika virus infection

**DOI:** 10.1590/0100-3984.2016.0219

**Published:** 2018

**Authors:** Alexandre Ferreira da Silva

**Affiliations:** 1 Ecotomo S/S Ltda., Belém, PA, Brazil.

Dear Editor,

Congenital central nervous system infections are accompanied by pathological intracranial calcifications, and cerebral organogenesis malformations are common in viral infections, particularly when they occur in the first trimester of gestation^(1-5)^. Intracranial calcifications with brain malformations have been reported in cytomegalovirus infection, congenital rubella, and, more recently, in Zika virus infection^(1,2,4,5)^. In cases of congenital toxoplasmosis, calcifications are seen in 50-80% of cases and hydrocephalus is a common finding, although defects in organogenesis induced by nonviral etiologic agents are rare^(3,4)^.

In the neonatal period, the diagnosis of congenital cytomegalovirus infection can be simple in a child presenting with fever, jaundice, hepatosplenomegaly, anemia, thrombocytopenia, and retinopathy. In cases of Zika virus infection, the central clinical aspect is microcephaly^(2,3,6,7)^. In congenital cytomegalovirus infection, the characteristic presentation is brain calcifications. Those calcifications are often periventricular, in the ependymal or subependymal region, appearing as points or lines or, in some cases, delineating the ventricles. The calcification foci, which can occur in the basal ganglia, white matter, or cortex, are often asymmetric^(1-5)^.

**Figure 1:**
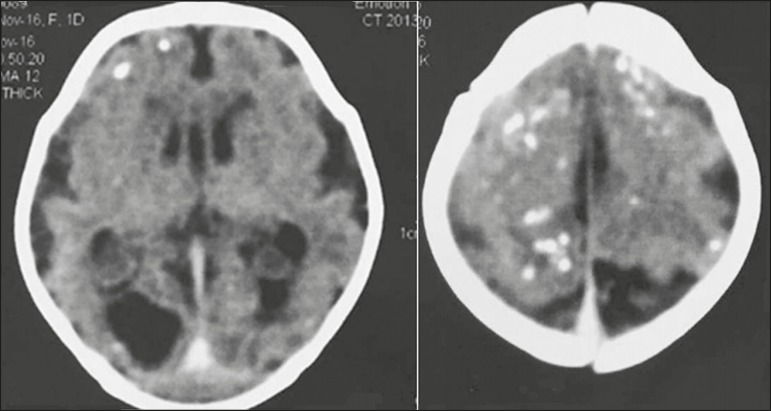
Non-contrast-enhanced computed tomography of the brain in a one-day-old neonate with lesions attributed to Zika virus infection, showing subcortical pathological intracranial calcifications and microcephaly with a compromised aspect of the cerebral sulci, malformation of the opercula, marked reduction in the cerebral white matter volume, and ventricular dilation.

Although congenital rubella is exceptionally rare in Brazil, some cases have been reported. The radiological findings are similar to those of cytomegalovirus infection. White matter anomalies and periventricular calcifications are often present, as are calcifications in the basal ganglia^(4)^. Unlike other congenital viral infectious processes associated with encephalic malformations, in which the distribution is typically periventricular, the Zika virus appears to produce subcortical calcifications (Figure 1).

**Figure 2:**
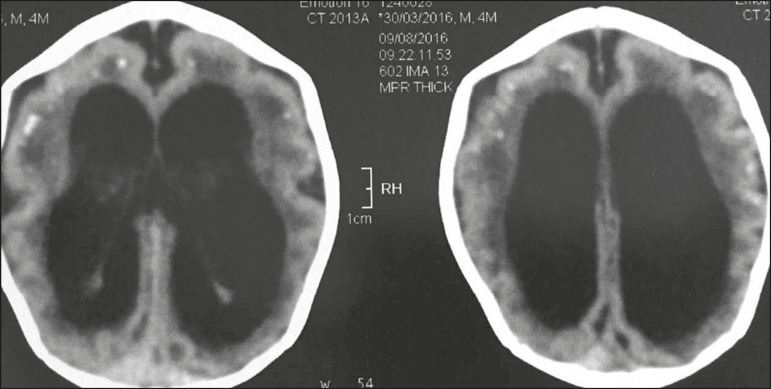
Non-contrast-enhanced computed tomography of the brain in a four-month-old child with lesions attributed to Zika virus infection, showing pathological subcortical intracranial calcifications,microcephaly, and ventricular dilatation, with simplification of the cerebral convolutions.

The association among intracranial calcifications, congenital infections, and central nervous system malformations is broad and requires the observance of some aspects. Congenital microcephaly can be divided into two main categories: primary and secondary. Some patients with primary congenital microcephaly have been described as having congenitally small but architecturally normal brains, which does not occur in cases of microcephaly associated with diverticulum and cleavage malformations such as holoprosencephaly or cerebral cortical defects such as lissencephaly, usually associated with nonprogressive mental retardation of a presumed genetic cause. In contrast, in cases of microcephaly acquired as a result of brain damage, such as those associated with hypoxic-ischemic injury, congenital central nervous system infection, or metabolic disease, the head size can initially be normal but can decrease as a result of the brain injury. However, in cases of Zika virus infection, microcephaly and brain calcifications, with simplification of the cerebral convolutions, are present on the first day of life (Figure 2).

The causes of pathological intracranial calcifications in children are diverse. Nevertheless, the combination of microcephaly and defects of cerebral organogenesis, especially those related to impairment of neuronal migration, is a strong indication of congenital central nervous system infection with a viral agent, and subcortical predominance of calcifications should prompt the radiologist to consider the hypothesis of Zika virus infection.
